# Characterization of Yellow and Red Pigments Associated with Soybean Reddening During Processing

**DOI:** 10.3390/foods15173030

**Published:** 2026-08-27

**Authors:** Shiori Idogawa, Serina Imaruoka, Taiki Miyazawa, Ohki Higuchi, Kazuaki Akasaka, Teruo Miyazawa

**Affiliations:** 1 Taishi Foods Inc., 68 Aza-Okinaka, Kawamorita, Sannohe-machi, Sannohe-gun 039-0141, Aomori, Japan; 2 New Industry Creation Hatchery Center (NICHe), Tohoku University, Sendai 980-8579, Miyagi, Japan; 3 Department of Human Health and Nutrition, Shokei Gakuin University, 4-10-1 Yurigaoka, Natori 981-1295, Miyagi, Japan

**Keywords:** soybean reddening, pigment transformation, yellow pigment, red pigment, lipoxygenase, tofu, mass spectrometry

## Abstract

Discoloration of soybean-based foods, particularly reddening during processing, remains poorly understood. In this study, yellow and red pigments associated with soybean reddening were extracted, purified, and characterized. The pigments showed distinct UV–Vis spectra, with a shoulder near 420 nm for the yellow pigment and an absorption maximum near 510 nm for the red pigment. Heat and pH stability experiments showed that the yellow pigment decreased during thermal treatment, accompanied by an increase in the red pigment. Mass spectrometric analyses revealed a consistent 2 Da mass difference and a common fragment ion, indicating a close structural relationship between the pigments. Both pigments were detected in soybean, soymilk, and tofu, and the red pigment increased during processing. The pigments did not correspond to known major soybean constituents. These findings suggest that soybean reddening involves the conversion of a yellow pigment into a structurally related red pigment during soybean processing.

## 1. Introduction

Color is an important quality attribute that influences the perceived value and acceptability of foods, as consumers often assess freshness and quality based on visual appearance. Unexpected color changes during processing and storage can raise concerns regarding food safety and reduce product value. Previous studies have reported that consumers have expectations regarding the color of foods, and deviations from the expected appearance can negatively affect quality perception and acceptance [1,2]. In particular, for light-colored foods such as dairy products, soymilk, and tofu, unexpected discoloration may be recognized as a sign of quality deterioration or abnormality.

Food discoloration is caused by a variety of chemical reactions, including the degradation and transformation of natural pigments, lipid oxidation, amino-carbonyl reactions (Maillard reactions), and enzymatic oxidation. In soybean-based foods, lipid hydroperoxides and aldehydes generated through the oxidation of unsaturated fatty acids are known to react with amino compounds, leading to carbonyl-amino reactions and subsequent secondary reactions that produce colored compounds [3]. These reactions are also associated with the Maillard reaction and contribute to non-enzymatic browning [4]. In addition, lipid oxidation mediated by lipoxygenase (LOX) has been reported to be involved in discoloration phenomena such as fading, browning, and reddening in soybean-derived foods [5].

In recent years, studies on food color changes have expanded beyond major pigments to include minor phenolic compounds. Certain flavonoid compounds contribute to coloration, and their color characteristics are known to vary depending on chemical structure and reaction conditions [6]. Soybean products, particularly soymilk and tofu, are expected to exhibit a characteristic pale yellow color; however, the phenomenon of “reddening” during processing has long been recognized as a problem [5]. Although the reddening phenomenon has long been recognized during soybean processing, the pigments responsible for the characteristic red coloration have not been chemically identified, and the mechanisms underlying their formation remain poorly understood. Furthermore, the color of soymilk has been reported to be significantly affected by heating conditions [7], suggesting that the mechanisms of reddening involve complex chemical reactions that have not yet been fully elucidated.

Advances in high-resolution mass spectrometry, such as LC-ESI-QTOF-MS/MS, have enabled the comprehensive detection of trace phenolic compounds and their oxidation products formed during food processing [8]. Soybeans also contain various isoflavone glycosides, including daidzin and genistin and their malonyl derivatives, which are known to undergo demalonylation and hydrolysis under heating and oxidative conditions [9,10]. These transformation products may also be involved in color changes. Traditionally, the reddening phenomenon in soymilk has been interpreted as part of non-specific non-enzymatic browning associated with lipid oxidation [4]. However, the pigment investigated in this study has been difficult to separate from known soybean components, and it is highly unstable in an isolated state; therefore, its chemical structure and formation mechanism remain unclear. In addition, the content and composition of soybean isoflavones are known to vary depending on cultivar and cultivation conditions [11], which may influence pigment formation behavior.

In this study, we aimed to characterize previously unidentified yellow and red pigments associated with discoloration during soybean processing. The effects of pH, heating, and LOX-mediated oxidation on pigment transformation were investigated. Structural information on the pigments was obtained using LC-ESI-QTOF-MS/MS and possible reaction pathways were explored. Particular attention was given to the characteristic 2 Da mass difference observed between the yellow and red pigments, which provided insight into their structural relationship and possible transformation mechanism. In addition, differences in pigment behavior among soybean cultivars were comparatively analyzed.

## 2. Materials and Methods

### 2.1. Chemicals and Reagents

Hexane (analytical grade), isopropanol (analytical grade), methanol (analytical grade and HPLC grade), acetonitrile (HPLC grade), and acetic acid (analytical grade) were purchased from FUJIFILM Wako Pure Chemical Corporation (Osaka, Japan). LC/MS-grade ultrapure water was used for LC/MS analysis. Distilled water was prepared using a water purification system (RFD240ND, ADVANTEC, Tokyo, Japan).

### 2.2. Materials

Soybean cultivar GL3500 (2020 harvest, Kanematsu Corporation, Tokyo, Japan; KG Agri Products, Inc., Marysville, OH, USA) was used in this study. GL3500 is a cultivar commonly used for tofu production, and preliminary experiments indicated a relatively high susceptibility to discoloration. Thus, it was selected as the experimental material. Additional soybean and soybean-derived samples from different cultivars and harvest years were used for the experiments described in the following sections.

The target yellow pigment has been reported to be a water-soluble, low-molecular-weight compound that passes through an ultrafiltration membrane with a molecular weight cutoff of 10,000 Da [12]. Therefore, extraction and purification procedures were designed based on these physicochemical properties.

### 2.3. Preparation of Discolored Tofu Samples

Soymilk was prepared using a production line at Taishi Foods Inc. (Miyagi, Japan) to obtain raw material for pigment extraction. The soymilk was heated at 101 °C for 2 min. The prepared soymilk was stored at 8 °C for 26 h, and then stirred for 1 min 30 s using a mixer (Bamix, ESGE AG, Mettlen, Switzerland) to promote oxidation. Subsequently, an aqueous solution of 25% magnesium chloride hexahydrate (MgCl_2_·6H_2_O) was added as a coagulant to achieve a final concentration of 0.4%, followed by additional stirring for 1 min 30 s. Approximately 5 L of soymilk was placed into a 10 L bag-in-box container and heated in a water bath (TBN402DA, ADVANTEC) at 85 °C for 1 h to induce coagulation. After cooling with water, tofu samples were obtained. The tofu samples were crushed, frozen at −80 °C, and freeze-dried (RLE series, Kyowa Vacuum Engineering Co., Ltd., Tokyo, Japan). Approximately 8.6 kg of freeze-dried powder was obtained from 60 kg of tofu.

### 2.4. Defatting Pretreatment

Defatting of the freeze-dried tofu was performed with modifications based on the method of Hara and Radin [13]. Freeze-dried tofu (150 g) was mixed with 1000 mL of hexane–2-propanol (3:2, *v*/*v*) and shaken at room temperature (160 rpm, 5 min) using a shaker (SHAKER MINI SS-80, EYELA, Tokyo, Japan). The mixture was then centrifuged at 25 °C and 10,000 rpm for 10 min (CR21N, rotor R10A3, HITACHI, Tokyo, Japan). After removal of the supernatant, the residue was subjected to the same extraction procedure twice more (total of three times). The defatted residue was air-dried at room temperature. Repetition of this procedure yielded approximately 1.0 kg of defatted powder from 1.2 kg of freeze-dried tofu.

### 2.5. Pigment Extraction

Defatted powder (100 g) was ground using an Oster blender, and 500 mL of 50% (*v*/*v*) methanol was added for extraction at room temperature. The extract was filtered under reduced pressure using qualitative filter paper No. 2 (ADVANTEC) with a Büchner funnel.

The residue was re-extracted with an additional 500 mL of 50% methanol, and the filtrates were combined. Methanol was removed under reduced pressure using a rotary evaporator (R-210, B-491, V-700, BÜCHI Labortechnik AG, Flawil, Switzerland).

### 2.6. Fractionation of Extract

The extract was purified using column chromatography. A glass column (30 mm × 500 mm) packed with porous styrene–divinylbenzene resin (SEPABEADS™ SP825L, Mitsubishi Chemical Corp., Tokyo, Japan) was equilibrated with methanol and distilled water. The extract was loaded onto the column, washed with distilled water, and eluted with 360 mL methanol. The eluate was concentrated under reduced pressure, dissolved in distilled water, and centrifuged to remove precipitates. The supernatant was further purified using DIAION™ HP2MG resin (Mitsubishi Chemical Corp.) under similar conditions and eluted with 400 mL methanol. The eluate was concentrated to obtain purified fractions. Approximately 1.0 g of pigment-enriched fraction was obtained from 100 g of starting material.

### 2.7. Sample Preparation

Purified pigment fraction (1.0 g) was dissolved in 1.5 mL of distilled water, filtered through a 0.45 μm PTFE syringe filter (ADVANTEC), and stored at −20 °C until use.

### 2.8. HPLC Systems

Two HPLC systems (preparative HPLC and analytical HPLC) were used.

Preparative HPLC (Method 1): The preparative HPLC system consisted of a PU-980 pump, CO-4060 column oven, RI-4030 refractive index detector, and AS-4050 autosampler (JASCO Corp., Tokyo, Japan), together with a 2487 UV–Vis detector (Waters Corp., Milford, MA, USA). Separation was performed using a YMC-Pack ODS-AM column (5 μm, 4.6 × 250 mm; YMC Co., Ltd., Kyoto, Japan) equipped with a guard column. The column temperature was maintained at 30 °C. The mobile phases consisted of 0.1% acetic acid in water (A) and 0.1% acetic acid in acetonitrile (B). The flow rate was 1.0 mL/min. The gradient profile was 12% B at 0 min, 25% B at 15 min, 35% B at 25 min, 100% B at 27 min and held until 32 min, returned to 12% B at 33 min, and equilibrated until 38 min. The detection wavelength was 460 nm.

Analytical HPLC (Method 2): The analytical HPLC system consisted of a DGU-20A degasser, LC-20AD pump, CTO-20A column oven, SPD-20A UV–Vis detector, and SIL-20AC autosampler (Shimadzu Corp., Kyoto, Japan). Separation was performed using a YMC-Triart C18 column (3 μm, 3.0 × 150 mm; YMC Co., Ltd.) equipped with a guard column. The column temperature was maintained at 30 °C. The mobile phases were the same as those used in Method 1. The flow rate was 0.4 mL/min. The gradient profile was 12% B at 0 min, 25% B at 15 min, 35% B at 25 min, 60% B at 27 min and held until 32 min, returned to 12% B at 33 min, and equilibrated until 38 min. Detection was performed at 420 and 500 nm.

### 2.9. Fractionation of Pigments

Pigment-enriched fraction (1.0 g) dissolved in water (1.0 mL) was fractionated using Method 1. A volume of 100 μL was injected per run, and the procedure was repeated 15 times. Because a delay occurred between UV detection and fraction collection due to the flow path between the detector and the collection point, the collection windows were adjusted relative to the observed chromatographic retention times. Fractions corresponding to the target peaks were manually collected at predetermined time windows of 22.3–22.8 min and 26.4–27.0 min. The collected fractions were freeze-dried before further analysis.

### 2.10. UV–Vis Spectroscopy

Freeze-dried pigments were dissolved in 1.0 mL of 70% ethanol. Spectra were recorded using a UV–Vis spectrophotometer (V-750, JASCO Corp.) with a 1 cm quartz cell. Conditions: 190–900 nm, absorbance mode, bandwidth 2.0 nm, response time 0.96 s.

### 2.11. pH Stability

Yellow pigment (0.5 mg) was dissolved in 0.5 mL phosphate buffer (pH 2–10, 100 mM). Samples were equilibrated at 30 °C for ≥2 h and heated at 80 °C for 90 min. Red pigment (0.3 mg) was dissolved in 0.6 mL of the same buffer to obtain a final concentration of 0.5 mg/mL and was treated similarly, except that the equilibration time at 30 °C was ≥3 h. Pigment levels before and after heating were analyzed by HPLC (Method 2).

### 2.12. Thermal Stability

Time dependence: Freeze-dried yellow pigment (2.0 mg) was dissolved in 2.0 mL of 100 mM potassium phosphate buffer (pH 6.5). After equilibration at 30 °C for 2 h, the solution was heated at 80 °C for up to 480 min. Pigment levels were quantified by analytical HPLC (Method 2).

Temperature dependence: Freeze-dried yellow pigment (2.0 mg) was dissolved in 2.0 mL of 100 mM potassium phosphate buffer (pH 6.5). After equilibration at 30 °C for 2 h, aliquots (250 μL) were transferred into 3 mL sealed vials and heated at 40–94 °C for 30 min. Pigment levels were quantified by analytical HPLC (Method 2).

### 2.13. Statistical Analysis

Experiments were performed independently three to five times for the pH stability study and three to four times for the thermal stability study. Results are presented as the mean ± standard deviation (SD).

For the pH stability and thermal stability studies, statistical analyses were per-formed using one-way analysis of variance (ANOVA) followed by Tukey’s multiple comparison test. Differences were considered statistically significant at *p* < 0.05. For experiments with n = 2, inferential statistical analyses were not performed, and the results are presented descriptively.

### 2.14. Relative Pigment Content in Soybean Materials

Soybean samples used for pigment analysis included Miyagi Shirome (2023 and 2024 harvests; Miyagi, Japan), Yukihomare (2022 and 2023 harvests; Hokkaido, Japan), and GL3500 (2024 harvest; Kanematsu Corporation). Soybeans (15 g) were soaked in deionized water (60 g) for 20 h. After soaking, deionized water was added to adjust the total weight of the soybeans and water to 90 g, and the soybeans were ground for 1 min using a blender (GRIND mode, Oster, Boca Raton, FL, USA). The resulting slurry was sealed in a plastic bag, heated in a constant-temperature water bath (TC-201D, Brookfield Engineering Laboratories, Middleboro, MA, USA) at 95 °C for 10 min, and then cooled with water. The slurry was centrifuged using a high-speed refrigerated centrifuge (CR21N, rotor R18A, HITACHI), and the supernatant was collected as soymilk. Tofu was prepared by adding 163 µL of 25% magnesium chloride solution to 10 g of soymilk to obtain a final MgCl_2_ concentration of 0.4%, followed by coagulation at 85 °C for 1 h. Raw soybeans were ground into soybean powder using an ultracentrifugal mill (ZM-200, Retsch GmbH, Haan, Germany) at 16,000 rpm. Soymilk and tofu were freeze-dried using a freeze dryer (FDU-1100, DRC-1N, EYELA).

Soybean, soymilk, and tofu samples were prepared and analyzed. Pigment extraction was carried out using 50% methanol. Briefly, 50% methanol (0.5 mL) was added to each sample (0.1 g), followed by vortex mixing and extraction at 120 rpm for 30 min at room temperature using a shaker (EYELA, SHAKER MINI SS-80). The extracts were centrifuged at 10,000 rpm for 10 min at 25 °C, and the supernatants were analyzed by HPLC (Method 2) to determine the relative amounts of the yellow and red pigments. Relative pigment content was calculated as peak area × extraction solvent volume (mL)/sample weight (g)/solid content (%)/injection volume (µL). Results are presented as mean ± SD (n = 2).

### 2.15. Lipoxygenase Treatment

Soymilk prepared from Miyagi Shirome (2023 harvest) and GL3500 (2024 harvest) soybeans using the procedure described in the previous section was used for the lipox-ygenase treatment experiments. Soymilk samples (3.0 mL) were treated with lipoxygenase (Lipoxidase from Soybean; Tokyo Chemical Industry Co., Ltd., Tokyo, Japan) under conditions listed in Table 1 and incubated at 25 °C. Some samples were heated at 80 °C for 30 min. Samples were freeze-dried, extracted, and analyzed as described above. Relative pigment content was calculated using the same equation. Results are presented as mean ± SD (n = 2).

### 2.16. Mass Spectrometric Analysis

The yellow and red pigment fractions obtained by preparative HPLC were analyzed by LC-ESI-QTOF-MS/MS (SCIEX LC system coupled with X500R QTOF-MS/MS, SCIEX, Marlborough, MA, USA).

Chromatographic separation was performed using a YMC-Triart C18 column (3 μm, 3.0 mm i.d. × 150 mm; YMC Co., Ltd.). The mobile phases consisted of 0.1% formic acid in water (solvent A) and 0.1% formic acid in acetonitrile (solvent B). Gradient elution was carried out at a flow rate of 0.4 mL/min. The gradient program was as follows: 17.5% B from 0 to 3.75 min, 20% at 20 min, 25% at 25 min, 31% at 35 min, 40% from 38 to 41 min, and returned to 17.5% at 44 min. The injection volume was 2 μL. The column temperature was maintained at 35 °C, and the autosampler temperature was set at 4 °C.

Mass spectrometric analysis was performed using electrospray ionization (ESI) in both positive and negative ion modes. MS/MS spectra were acquired using the information-dependent acquisition (IDA) method. The mass range was set to *m*/*z* 50–1500, and collision-induced dissociation was applied to selected precursor ions.

In addition, selected ions from the yellow and red pigment fractions were further analyzed using a triple quadrupole/linear ion trap mass spectrometer (QTRAP 6500+ system, SCIEX, Marlborough, MA, USA). Ionization was performed using ESI in both positive and negative modes. Product ion spectra were obtained by collision-induced dissociation (CID), and fragmentation patterns of the yellow and red pigment fractions were compared. For comparison, soyasapogenol A, soyasapogenol B, and riboflavin were also analyzed under the same MS/MS conditions, and their spectra were compared with those obtained from the pigment fractions.

## 3. Results

### 3.1. Extraction and Purification of Pigments

**Figure 1:**
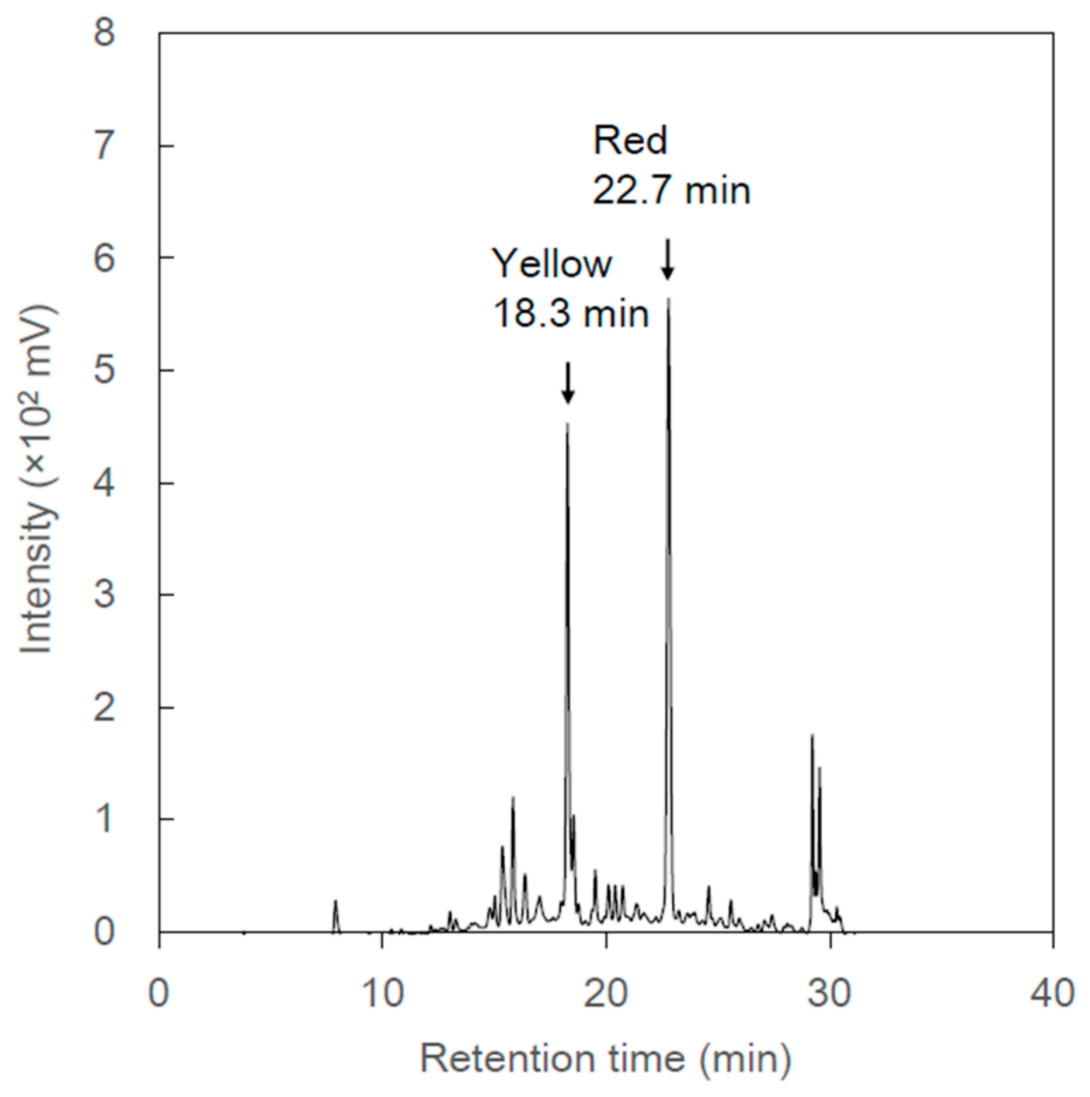
HPLC chromatogram of crude pigment extract from tofu obtained using Method 1 (UV detection at 460 nm), showing separation of the yellow and red pigments.

Pigment fractions extracted from the raw material were purified using adsorption resin chromatography and further separated into yellow and red pigments using HPLC Method 1. The chromatogram obtained during fractionation is shown in Figure 1. The retention times (Rt) of the yellow and red pigments in HPLC Method 1 were 18.3 min and 22.7 min, respectively.

### 3.2. Characterization of Pigments

**Figure 2:**
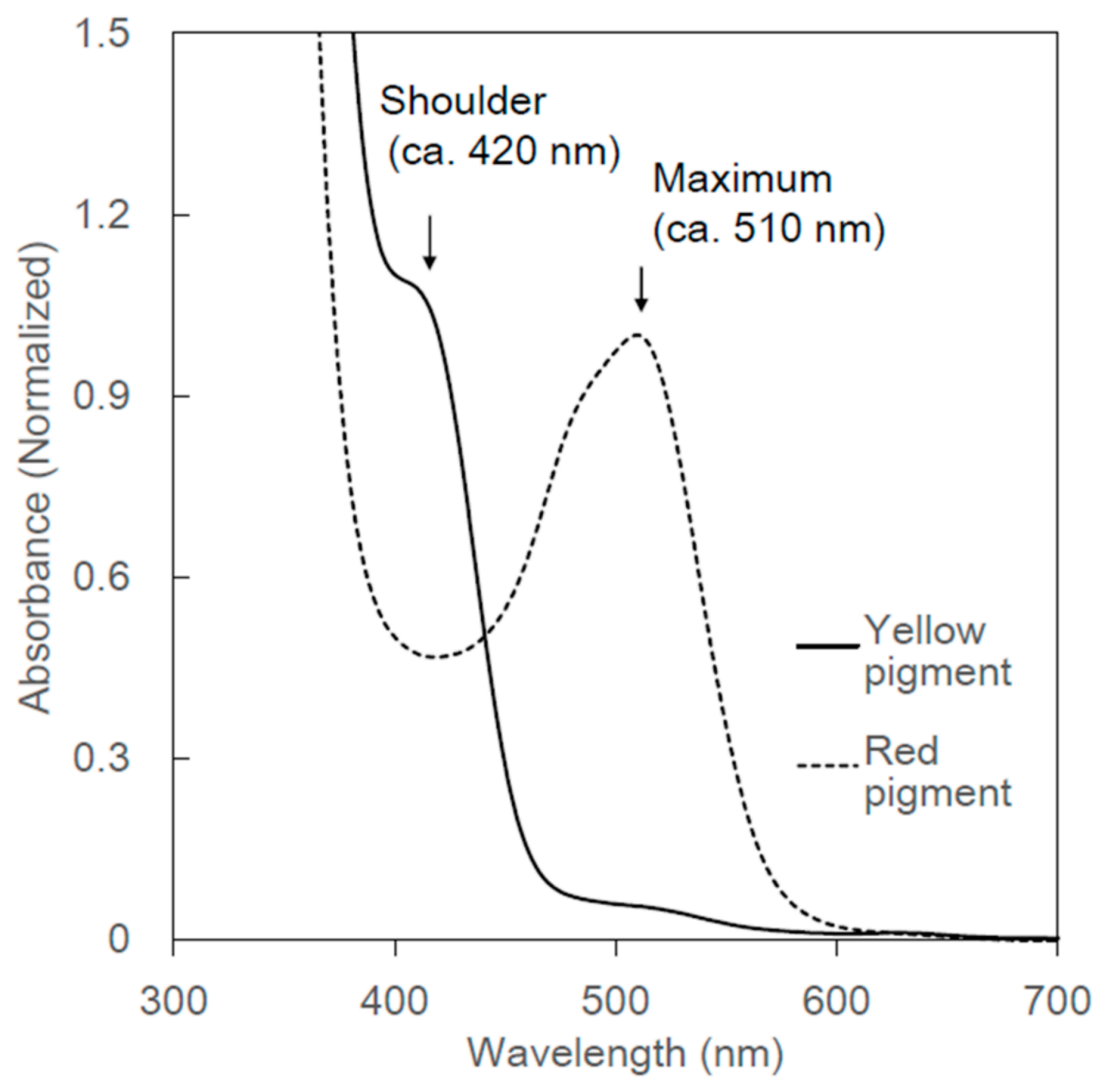
UV–Vis absorption spectra of purified yellow and red pigments. The yellow and red pigment spectra were normalized to the absorbance at approximately 420 and 510 nm, respectively. The yellow pigment shows a shoulder at around 420 nm, whereas the red pigment exhibits an absorption maximum at approximately 510 nm.

**Figure 3:**
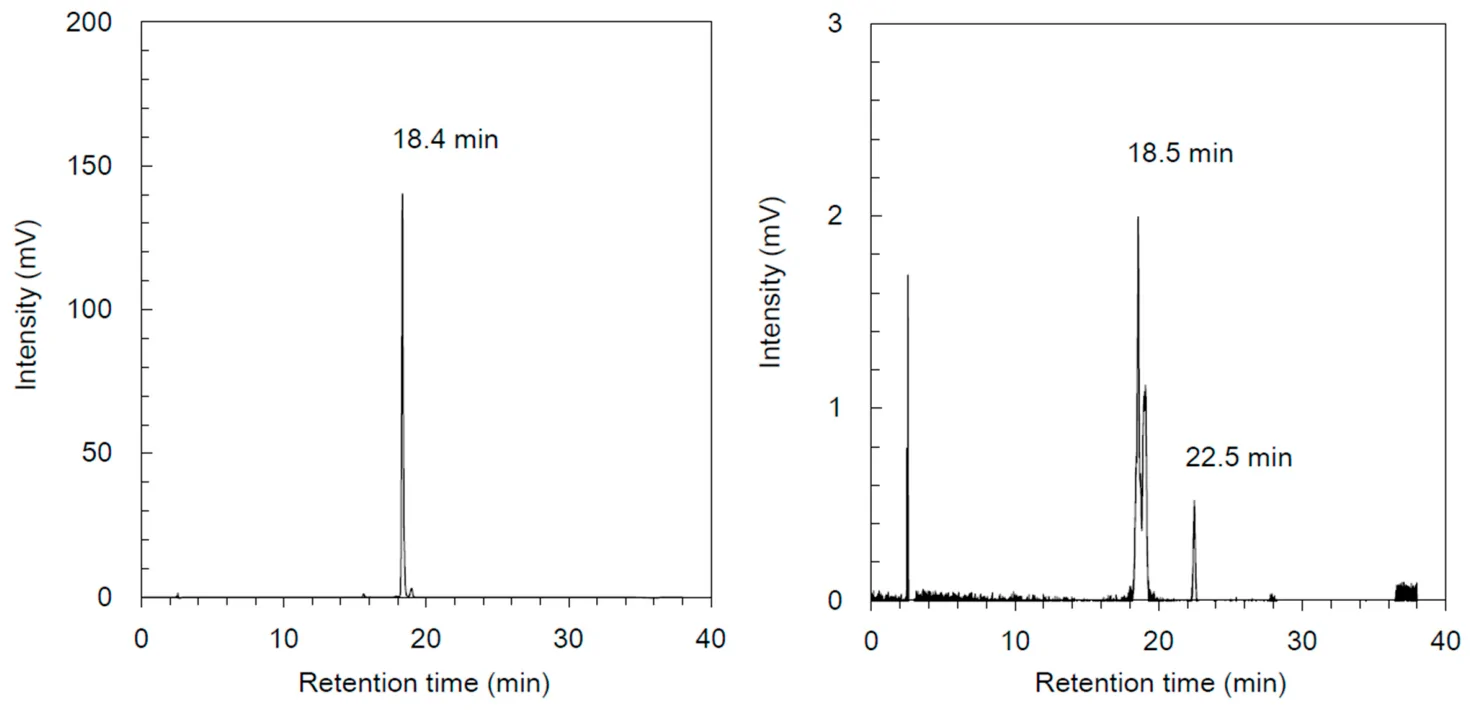
HPLC chromatograms of purified pigments obtained using Method 2. (**Left**): yellow pigment detected at 420 nm; (**right**): red pigment detected at 500 nm. Note that the intensity scales differ between chromatograms.

The yellow and red pigments obtained by preparative HPLC (Method 1) were dissolved in 70% ethanol, and their UV–Vis absorption spectra were measured (Figure 2). To facilitate a comparison of spectral features, the spectra were normalized to the absorbance at approximately 420 nm for the yellow pigment and 510 nm for the red pigment. The yellow pigment exhibited a characteristic shoulder at approximately 420 nm and did not show a distinct absorption maximum in the visible region. In contrast, the red pigment showed a clear absorption maximum at approximately 510 nm. The absorption maximum of the red pigment was red-shifted by approximately 90 nm compared with the shoulder of the yellow pigment. Accordingly, wavelengths of 420 nm and 500 nm were selected as HPLC detection wavelengths for the quantitative analysis of the yellow and red pigments, respectively. Re-analysis of each fraction using HPLC Method 2 is shown in Figure 3. The retention times of the yellow and red pigments were 18.4 min and 22.5 min, respectively. In addition to the red pigment peak, a peak corresponding to the yellow pigment was detected in the red pigment fraction.

### 3.3. Effect of pH on Pigment Stability

**Figure 4:**
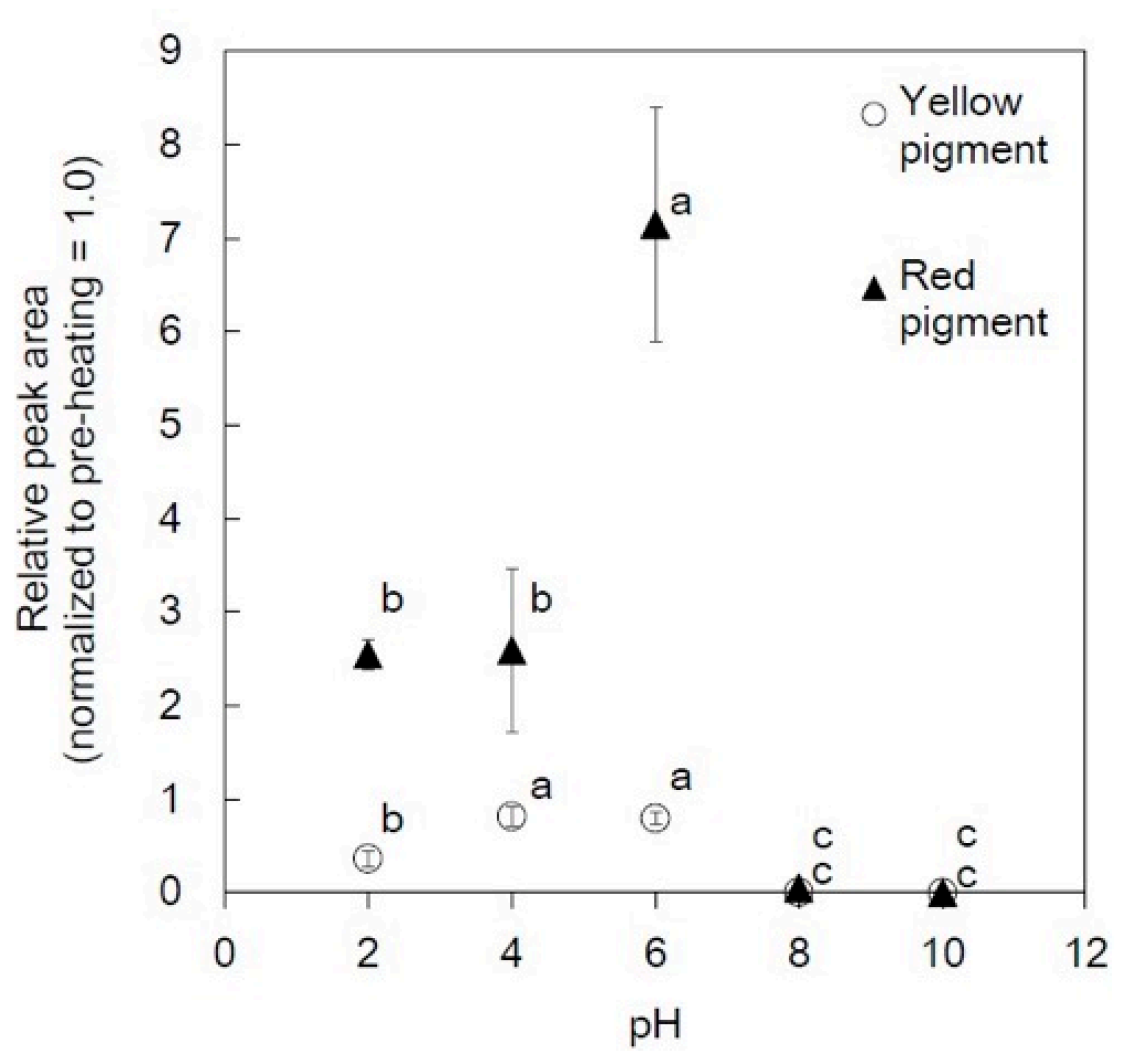
Effects of pH on the relative peak areas of yellow and red pigments after heating. Values were normalized to the peak area of the yellow pigment before heating (set to 1.0). Data represent mean ± SD (n = 3–5). Different lowercase letters indicate significant differences among pH conditions (one-way ANOVA followed by Tukey’s multiple comparison test, *p* < 0.05).

Figure 4 shows the relative peak areas of the yellow and red pigments after heating, normalized to the peak area of the yellow pigment before heating (set to 1.0) under each pH condition.

In the yellow pigment fraction, a small peak corresponding to the red pigment was also detected. After heating at 80 °C, the peak area of the yellow pigment decreased markedly at pH 8 and pH 10, and also decreased substantially at pH 2, whereas the decrease was less pronounced at pH 4 and pH 6. The peak areas at pH 4 and pH 6 were significantly higher than those at pH 2, pH 8, and pH 10 (Tukey’s test, *p* < 0.05).

The peak area of the red pigment was highest at pH 6, and increases were also observed at pH 2 and pH 4. The peak area at pH 6 was significantly higher than those at pH 2, pH 4, pH 8, and pH 10 (Tukey’s test, *p* < 0.05). However, the red pigment peak was barely detected under alkaline conditions (pH 8 and pH 10).

**Figure 5:**
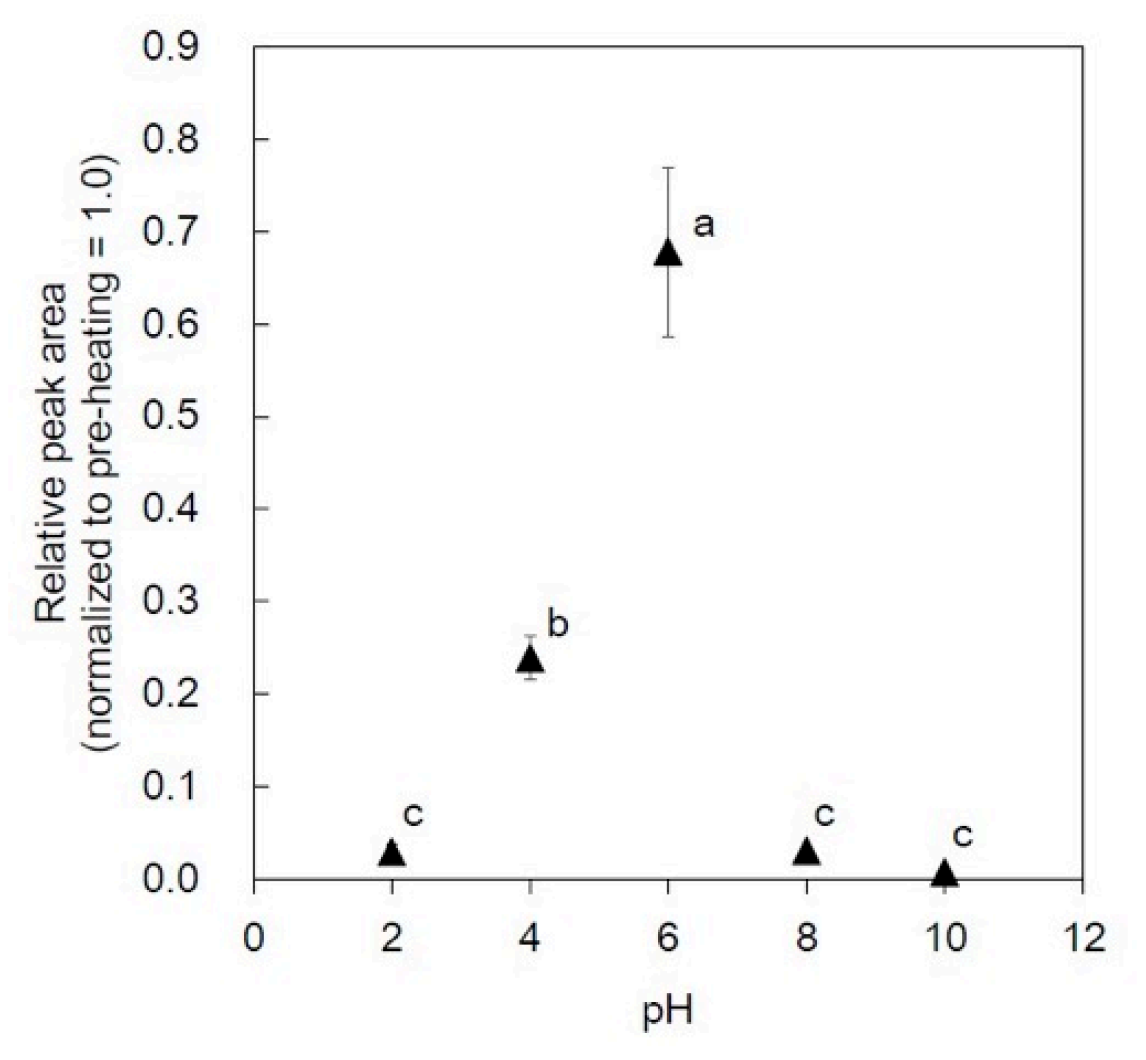
Effects of pH on the relative peak area of the red pigment after heating. Values were normalized to the peak area before heating (set to 1.0). Data represent mean ± SD (n = 3–4). Different lowercase letters indicate significant differences among pH conditions (one-way ANOVA followed by Tukey’s multiple comparison test, *p* < 0.05).

Figure 5 shows the relative peak areas of the red pigment after heating, normalized to the peak area before heating (set to 1.0). The red pigment peak area decreased markedly at pH 8 and pH 10, and a substantial decrease was also observed at pH 2. In contrast, relatively high values were maintained at pH 4 and pH 6. The peak area at pH 6 was significantly higher than those at all other pH conditions, and the peak area at pH 4 was significantly higher than those at pH 2, pH 8, and pH 10 (Tukey’s test, *p* < 0.05).

### 3.4. Effect of Heating on Pigment Stability

**Figure 6:**
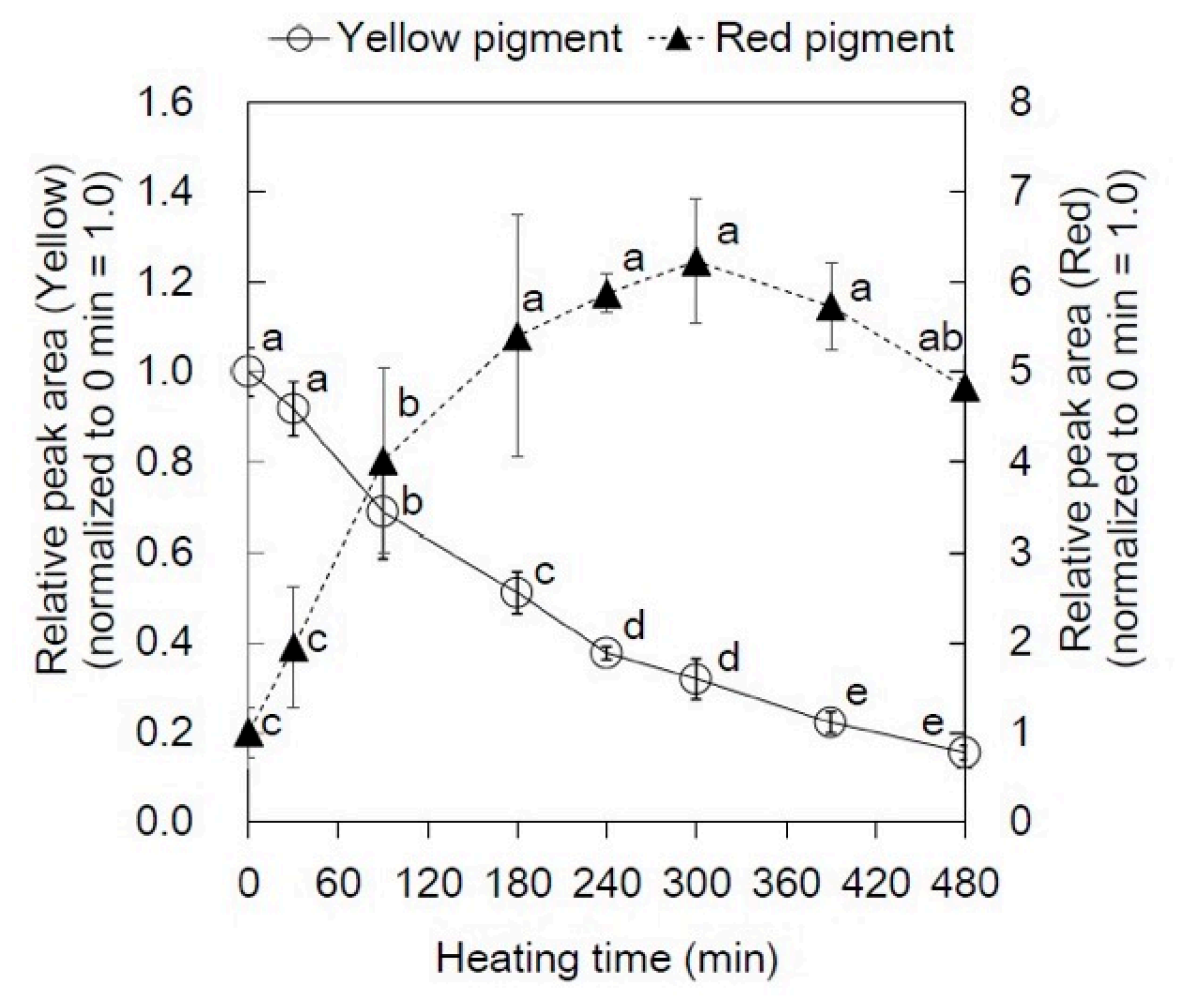
Changes in the relative peak areas of yellow and red pigments during heating at 80 °C. Values were normalized to the peak area at 0 min (set to 1.0). Data represent mean ± SD (n = 4). Different lowercase letters indicate significant differences among heating times (one-way ANOVA followed by Tukey’s multiple comparison test, *p* < 0.05).

Figure 6 shows the changes in the relative peak areas of the yellow and red pigments during heating at 80 °C, normalized to the values before heating (set to 1.0). The peak area of the yellow pigment decreased continuously with increasing heating time, and this decrease persisted up to 480 min. Significant differences were observed among heating times (Tukey’s test, *p* < 0.05). In contrast, the red pigment was detected in small amounts before heating and increased during heating, reaching a maximum at approximately 300 min, after which it began to decrease. The red pigment levels at 180–390 min were significantly higher than those at 0 and 30 min (Tukey’s test, *p* < 0.05).

**Figure 7:**
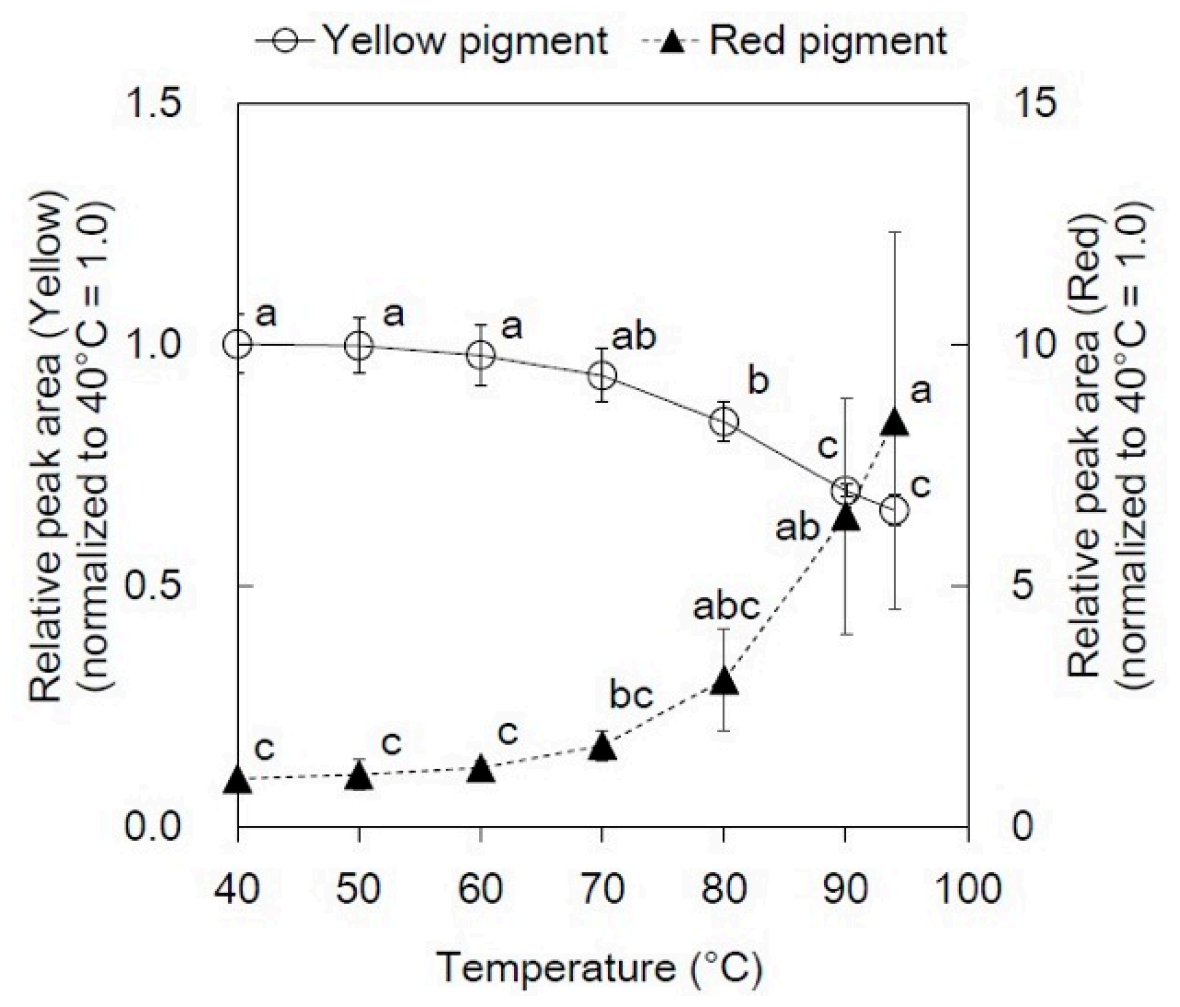
Effects of temperature on the relative peak areas of yellow and red pigments during heating. Values were normalized to the peak area at 40 °C (set to 1.0). Data represent mean ± SD (n = 3). Different lowercase letters indicate significant differences among temperatures (one-way ANOVA followed by Tukey’s multiple comparison test, *p* < 0.05).

Figure 7 shows the relationship between heating temperature and the relative peak areas of the yellow and red pigments, normalized to the values obtained at 40 °C (set to 1.0). The peak area of the yellow pigment showed little change up to 50 °C; however, it decreased above 60 °C and continued to decline with increasing temperature. The yellow pigment levels at 90 and 94 °C were significantly lower than those at 40–60 °C (Tukey’s test, *p* < 0.05). In contrast, the peak area of the red pigment increased as the temperature increased. The red pigment levels at 90 and 94 °C were significantly higher than those at 40–60 °C (Tukey’s test, *p* < 0.05).

### 3.5. Relative Pigment Contents in Soybean, Soymilk, and Tofu

**Figure 8:**
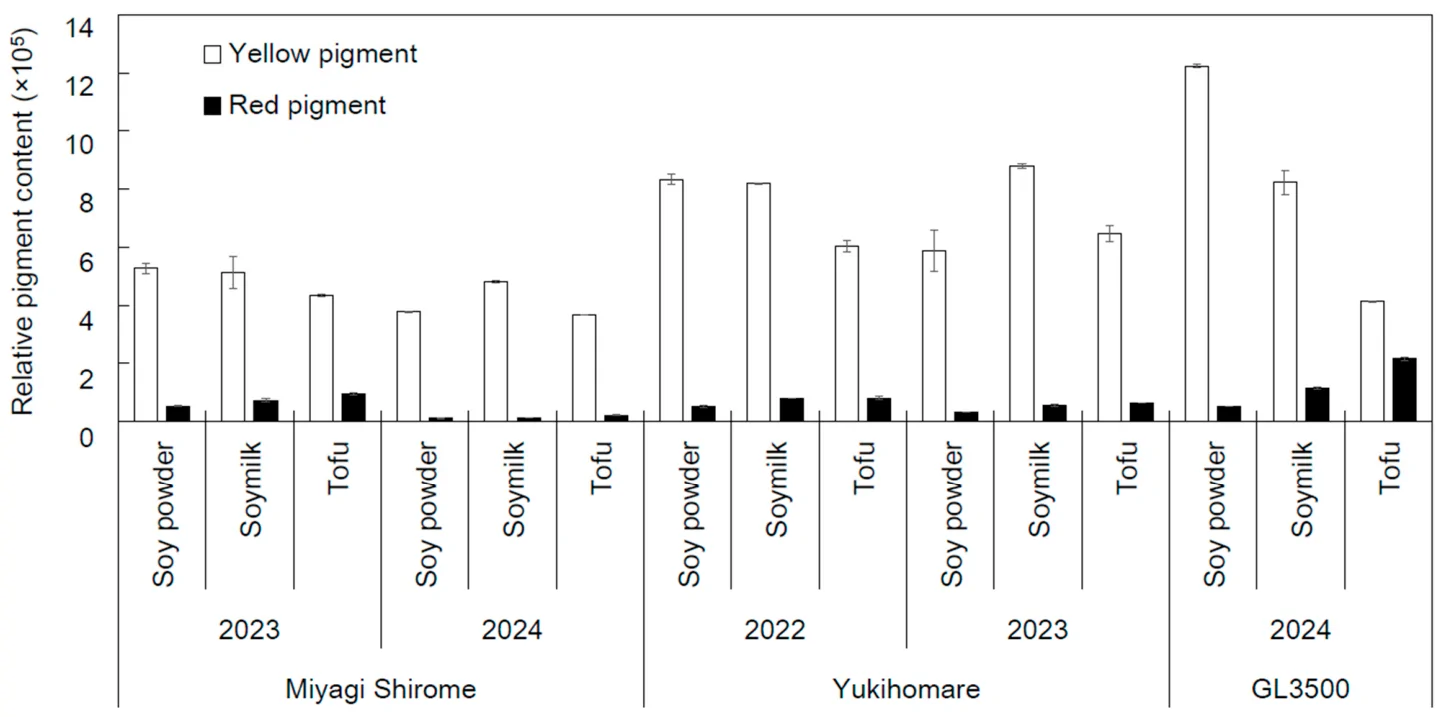
Relative contents of yellow and red pigments in soybean powder, soymilk, and tofu samples from different soybean cultivars and harvest years. Data represent mean ± SD (n = 2).

Figure 8 shows the relative pigment contents of the yellow and red pigments in soybean powder, soymilk, and tofu samples. Both pigments were detected in all samples. The red pigment content showed an increasing trend in the order of soybean powder < soymilk < tofu. Within the same cultivar, Miyagi Shirome (2023 harvest) exhibited a higher red pigment content than Miyagi Shirome (2024 harvest), with an approximately 4.6-fold difference in tofu samples. In addition, relatively high levels of red pigment were observed in GL3500. On the other hand, no clear correlation was observed between the contents of the yellow and red pigments. Samples with higher amounts of the yellow pigment did not necessarily show higher levels of the red pigment.

### 3.6. Effects of Lipoxygenase (LOX) on Relative Pigment Contents

**Figure 9:**
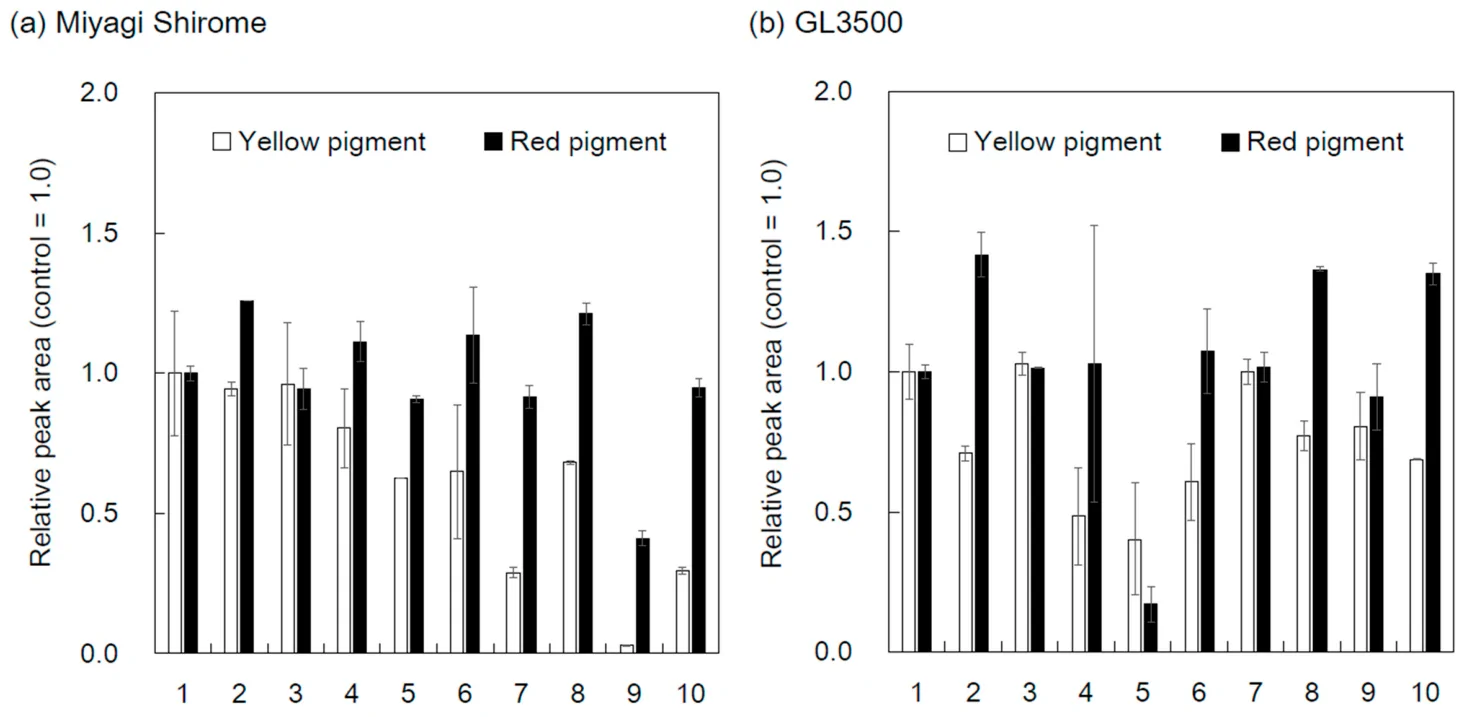
Effects of lipoxygenase addition and heat treatment on the relative peak areas of yellow and red pigments. Values were normalized to the control (set to 1.0). (**a**) Miyagi Shirome; (**b**) GL3500. Numbers 1–10 correspond to the treatment conditions listed in Table 1. Data represent mean ± SD (n = 2).

Figure 9 shows the effects of lipoxygenase (LOX) addition and heat treatment on the relative contents of the yellow and red pigments.

In Miyagi Shirome, the yellow pigment content tended to decrease with increasing LOX concentration and longer reaction time. Under conditions with high LOX concentration and longer reaction time (conditions 9 and 10), the lowest levels of the yellow pigment were observed. In contrast, the red pigment content was comparable to or slightly higher than that of the control under most conditions, but decreased under prolonged treatment with high LOX concentration.

In GL3500, the yellow pigment content decreased more prominently under heating conditions compared to LOX treatment alone. In contrast, the red pigment content was relatively high under heating conditions, particularly in samples subjected to heating after LOX treatment (conditions 8 and 10).

### 3.7. Analysis of Pigment Components

LC-ESI-QTOF-MS analysis revealed molecular-related ion peaks at *m*/*z* 665 and 663 for the yellow and red pigments, respectively, in positive ion mode, and at *m*/*z* 663 and 661, respectively, in negative ion mode. Thus, a consistent 2 Da mass difference between the yellow and red pigments was observed in both ionization modes.

**Figure 10:**
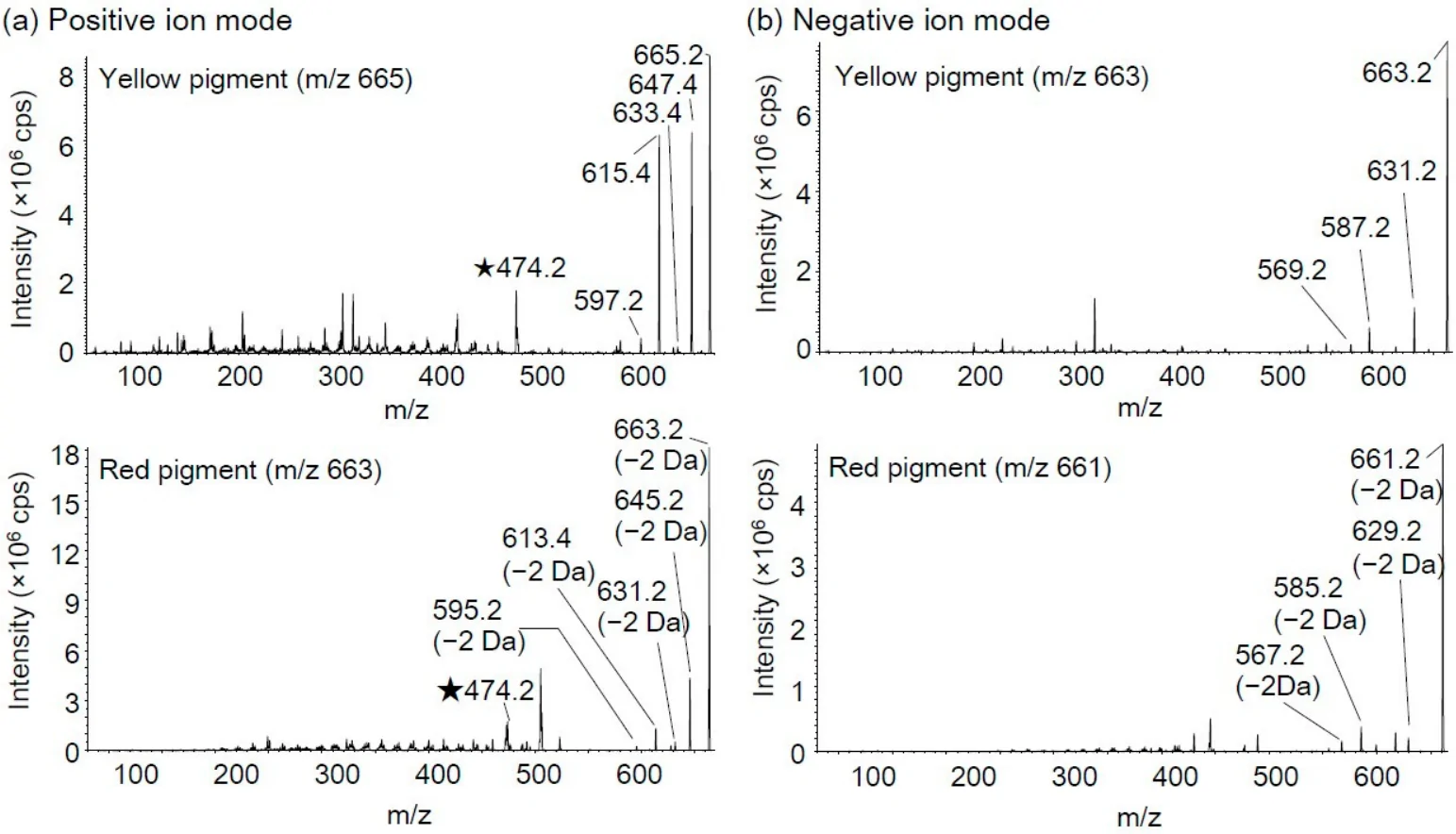
Product ion spectra of the yellow and red pigments obtained by QTRAP-MS/MS in (**a**) positive and (**b**) negative ion modes. Several corresponding fragment ions exhibited a consistent 2 Da mass difference between the pigments. The star symbol (★) indicates the fragment ion at *m*/*z* 474.2. The observed patterns support the presence of a closely related core structure in the yellow and red pigments.

Further MS/MS analysis using a QTRAP system (Figure 10) provided additional structural information. In positive ion mode, a fragment ion at *m*/*z* 474.2 was observed, together with several corresponding fragment ions showing a 2 Da mass difference between the yellow and red pigments. In negative ion mode, corresponding fragment ions with a 2 Da mass difference were also observed, although the number and distribution of the fragment ions differed between the two ionization modes.

The fragmentation pattern of the yellow pigment isolated from tofu was consistent with that of the yellow pigment detected in soybean powder. In contrast, the red pigment was scarcely detected in soybean powder. MS/MS analyses of soyasapogenol A, soyasapogenol B, and riboflavin, which have molecular weights similar to that of the yellow pigment, showed no matching fragmentation patterns. In addition, no soybean saponins with matching molecular weights and fragmentation patterns were identified in this study. Major soybean isoflavones, including daidzin, genistin, glycitin, and their malonyl derivatives, have molecular weights substantially lower than those of the pigments detected in this study. Furthermore, molecular formula estimation based on high-resolution mass spectrometry data and database searches did not identify any known soybean components corresponding to the observed pigments.

## 4. Discussion

### 4.1. Transformation of Yellow Pigment into Red Pigment

The present study provided evidence that the decrease in the yellow pigment was accompanied by the formation of a red pigment under thermal and oxidative conditions. During heating, the yellow pigment gradually decreased, whereas the red pigment increased and reached a maximum level before subsequently declining. The subsequent decline of the red pigment during prolonged heating suggests that the pigment itself is not a terminal product but may undergo further degradation or conversion into other compounds. In addition, the red pigment content tended to increase during tofu processing and under certain lipoxygenase (LOX) treatment conditions. These observations suggest that the red pigment is not an independently occurring compound but is formed from the yellow pigment through a specific transformation process. Previous studies interpreted the decrease in the yellow pigment during soybean processing primarily as a bleaching phenomenon associated with lipid oxidation mediated by lipoxygenase [12]. Lipoxygenase catalyzes the oxidation of polyunsaturated fatty acids, generating hydroperoxides and various secondary oxidation products [14,15], and has been implicated in quality deterioration and color changes in soybean-based foods [16,17]. In the present study, however, the decrease in the yellow pigment was accompanied by the appearance of a specific red pigment rather than by complete disappearance of the chromophore. This finding suggests that the yellow pigment may undergo chemical modification during processing instead of simple degradation. Thermal processing is known to induce oxidation, degradation, and the structural transformation of food constituents, often resulting in changes in color and functionality [18].

The pH stability experiments provided additional evidence for the conversion of the yellow pigment into the red pigment. Formation of the red pigment was most pronounced under weakly acidic to neutral conditions, particularly around pH 6, whereas little red pigment was detected under alkaline conditions. Moreover, the red pigment itself showed relatively high stability at pH 4–6 but decreased markedly at pH 8 and 10. These results suggest that both pigment formation and stability are strongly influenced by pH. The pH range in which the red pigment exhibited relatively high stability corresponds approximately to the typical pH range of tofu, suggesting that the pigment can persist during tofu production and storage [19]. Because tofu typically exhibits a pH around 6, these conditions may favor the accumulation and persistence of the red pigment during processing.

Mass spectrometric analyses further supported a close structural relationship between the two pigments. LC-ESI-QTOF-MS revealed a consistent 2 Da difference between the molecular-related ions of the pigments, while QTRAP-MS/MS showed a fragment ion at *m*/*z* 474.2 in positive ion mode and corresponding fragment ions exhibiting the same mass difference between the pigments. Such a mass difference is consistent with the loss of two hydrogen atoms and may be associated with oxidation or dehydrogenation reactions that increase molecular unsaturation. Oxidation-related structural modifications are commonly detected as characteristic mass shifts in mass spectrometric analyses of biological and food-derived compounds [20,21] and are frequently involved in the transformation of food-derived polyphenolic and protein-associated compounds during processing [22]. Furthermore, the fragmentation patterns indicated that both pigments retain a closely related core structure, suggesting that only a limited structural modification occurs during conversion.

Additional evidence for structural alteration was obtained from UV–Vis spectroscopy. The absorption maximum shifted from approximately 420 nm in the yellow pigment to approximately 510 nm in the red pigment. Bathochromic shifts of this magnitude are commonly associated with increased conjugation length or changes in the electronic structure of chromophoric systems [23,24,25]. Therefore, the observed spectral shift is consistent with the formation of a more highly conjugated chromophore in the red pigment.

A peak corresponding to the yellow pigment was also detected in the red pigment fraction (Figure 3), indicating that complete chromatographic separation of the two pigments was not achieved. In addition, both pigments were chemically unstable and gradually changed during purification and storage. These factors prevented reliable determination of their precise purity. Therefore, the collected fractions are more appropriately described as pigment-enriched fractions rather than purified pigments. The fractions were enriched in the respective target pigments, although co-eluting components likely remained.

Taken together, the thermal stability, LOX treatment, UV–Vis, and MS/MS results support the hypothesis that the yellow pigment serves as a precursor of the red pigment and undergoes a structural transformation during soybean processing. Although the exact chemical structure remains unknown, the conversion appears to involve oxidation-related modification accompanied by changes in molecular conjugation rather than simple pigment degradation.

A limitation of the present study is that the relationship between red pigment ac-cumulation and the macroscopic color characteristics of soybean-derived products was not quantitatively evaluated. The red pigment was detected in samples associated with soybean reddening during processing; however, visible color changes are likely influenced by multiple pigments and other compositional factors. Therefore, the red pigment should be regarded as a compound associated with soybean reddening rather than the sole determinant of the observed color change. Future studies combining objective color measurements such as CIELAB a* values and ΔE, and quantitative pigment analysis will be necessary to establish the relationship between red pigment accumulation and visible reddening in soybean, soymilk, and tofu.

### 4.2. Occurrence of Pigments in Soybean Materials

Both the yellow and red pigments were detected in soybean powder, soymilk, and tofu, indicating that these pigments are widely distributed in soybean-derived materials. The presence of the yellow pigment in soybean powder suggests that it originates from the soybean seed itself and serves as a precursor compound throughout soybean processing. In contrast, the relative content of the red pigment increased from soybean powder to soymilk and further to tofu, suggesting that red pigment formation proceeds during processing steps involving grinding, heating, and coagulation. Similar oxidation-related color development during processing has been reported in other food systems, where oxidation products contribute to progressive color changes [26]. Differences in pigment contents were also observed among cultivars and harvest years. In particular, Miyagi Shirome harvested in 2023 exhibited higher red pigment levels than the same cultivar harvested in 2024, especially in tofu samples. Although the factors responsible for these differences remain unclear, variations in soybean composition associated with cultivar, growing environment, and postharvest storage may contribute to pigment formation. Previous studies have shown that soybean phytochemical composition varies depending on cultivar and cultivation conditions [11], while storage can alter the composition and stability of soybean constituents through oxidative and other chemical reactions [27,28]. Such differences may influence either the amount of precursor compounds or the susceptibility of the pigments to transformation during processing. Notably, no clear relationship was observed between the amounts of the yellow and red pigments among the tested materials. This finding suggests that red pigment accumulation is not determined solely by the initial concentration of the yellow pigment. Instead, the efficiency of pigment conversion during processing, as well as cultivar-dependent differences in chemical composition and oxidative behavior, may play important roles in determining the final pigment profile. Such cultivar-dependent differences may partly explain the distinct responses to LOX treatment observed in the present study.

### 4.3. Possible Involvement of Lipoxygenase and Lipid Oxidation

Previous studies have suggested that the decrease in yellow pigment during soybean processing is associated with lipid oxidation mediated by lipoxygenase (LOX) [12]. Lipoxygenase catalyzes the oxidation of polyunsaturated fatty acids, generating hydroperoxides and a variety of secondary oxidation products [29,30]. These oxidation products are known to contribute to changes in flavor and color in soybean-based foods [16,17]. Formation of volatile oxidation products, such as n-hexanal, has been shown to be closely associated with lipoxygenase activity in soybean homogenates [31]. In the present study, the yellow pigment decreased during both LOX treatment and thermal processing, whereas the red pigment increased under several experimental conditions. However, the response differed depending on soybean cultivar. In Miyagi Shirome, the yellow pigment decreased with increasing LOX concentration and reaction time, suggesting a possible association between enzymatic oxidation and pigment transformation. In contrast, in GL3500, heat treatment had a greater effect on pigment behavior than LOX treatment alone, and elevated red pigment levels were observed particularly after heating. These findings suggest that transformation of the yellow pigment is influenced by oxidative conditions, although the contribution of LOX appears to vary among cultivars. Differences in LOX isozyme composition and activity have been reported among soybean cultivars [32], which may partly explain the cultivar-dependent responses observed in this study. Furthermore, lipid oxidation can proceed not only through LOX-catalyzed reactions but also through non-enzymatic pathways accelerated by heat [33]. The increase in red pigment observed during heating experiments supports the possibility that thermal oxidation contributes to pigment transformation. Taken together, these results suggest that oxidation plays an important role in the conversion of the yellow pigment into the red pigment, although the relative contributions of enzymatic and non-enzymatic pathways remain unclear. Further studies will be required to determine whether LOX is directly involved in the formation of the red pigment or whether it primarily promotes oxidative conditions that facilitate the transformation reaction.

### 4.4. Structural Characteristics and Possible Identity of the Pigments

Mass spectrometric analyses provided evidence that the yellow and red pigments are structurally related compounds. LC-ESI-QTOF-MS analysis revealed a consistent mass difference of 2 Da between the molecular-related ions of the two pigments in both positive and negative ion modes. In addition, MS/MS analysis using a QTRAP system demonstrated the presence of a fragment ion at *m*/*z* 474.2 in positive ion mode, together with several corresponding fragment ions that retained the same 2 Da mass difference. These results suggest that the yellow and red pigments share a closely related core structure and differ by a relatively small structural modification. To assess the possible identity of the pigments, MS/MS spectra were compared with those of several known soybean constituents. However, soyasapogenol A, soyasapogenol B, and riboflavin did not exhibit fragmentation patterns consistent with those of the pigment fractions. Furthermore, previously reported fragmentation characteristics of soybean saponins analyzed by tandem mass spectrometry [34] did not match the molecular weights or fragmentation behavior observed in this study. Major soybean isoflavones, including daidzin, genistin, glycitin, and their malonyl derivatives, also possess substantially lower molecular weights than the pigments identified here. In addition, molecular formula estimation based on high-resolution mass spectrometric data and database searches failed to identify any known soybean component corresponding to the observed pigments.

Taken together, the available spectroscopic and mass spectrometric evidence indicates that the yellow and red pigments constitute a closely related pair of compounds that differ by a small structural modification. The consistent 2 Da mass difference, together with the related fragmentation patterns, suggests that the pigments may represent a redox-related pair of structurally analogous compounds. Furthermore, the approximately 90 nm bathochromic shift observed in the UV–Vis spectra is consistent with alteration of the chromophore structure and the development of a more extended conjugated electronic system [23,25]. Although their exact chemical structures remain unknown, the present results indicate that the pigments are distinct from previously reported major soybean constituents. NMR spectroscopic analysis was undertaken as part of the structural investigation; however, complete structural elucidation was not achieved, likely owing to the limited purity and apparent instability of the isolated pigments. Further studies will therefore be required to determine their chemical identities and clarify the reaction mechanisms responsible for pigment transformation during tofu processing.

## Figures and Tables

**Table 1:** Lipoxygenase treatment conditions.

No.	Sample Condition	Lipoxygenase Concentration (mg/mL)	Incubation at 25 °C	Heating at 80 °C
**1**	Untreated	0	−	−
**2**	No LOX added	0	−	30 min
**3**	No LOX added	0	2 h	−
**4**	No LOX added	0	2 h	30 min
**5**	LOX added (1 mg/mL)	1	30 min	−
**6**	LOX added (1 mg/mL)	1	30 min	30 min
**7**	LOX added (2 mg/mL)	2	30 min	−
**8**	LOX added (2 mg/mL)	2	30 min	30 min
**9**	LOX added (2 mg/mL)	2	2 h	−
**10**	LOX added (2 mg/mL)	2	2 h	30 min

## Data Availability

The raw data supporting the conclusions of this article will be made available by the authors on request.
